# Mineral Homeostasis and Depression: Implications for Prevention and Therapeutic Support—A Narrative Review

**DOI:** 10.3390/ijms26146637

**Published:** 2025-07-10

**Authors:** Zuzanna Majewska, Karolina Orywal

**Affiliations:** 1 Faculty of Pharmacy with the Division of Laboratory Medicine, Medical University of Bialystok, Waszyngtona 15A, 15-269 Bialystok, Poland; 2 Department of Biochemical Diagnostics, Faculty of Pharmacy with the Division of Laboratory Medicine, Medical University of Bialystok, Waszyngtona 15 A, 15-269 Bialystok, Poland

**Keywords:** depression, calcium, magnesium, iron, zinc, copper, selenium, iodine, mineral homeostasis, micronutrients, neuroprotection, neuroinflammation

## Abstract

Depression affects approximately 280 million people worldwide and is becoming increasingly prevalent, particularly among young people. Despite numerous studies on the pathogenesis of this disorder, many factors remain unclear. New data in the literature suggest that proper concentrations of essential macro- and micronutrients play an important role in maintaining mental health and that disturbances in the metabolism of mineral compounds may contribute to the development and progression of depressive disorders. Numerous clinical and epidemiological studies have shown that low concentrations of these elements are associated with impaired neurotransmitter activity, increased exposure to oxidative stress, and neuroinflammation, all of which may contribute to the onset or exacerbation of depression. Additionally, some macro- and micronutrients may contribute to metabolic and hormonal disorders, thereby exacerbating their impact on mood regulation. A comprehensive literature search of the PubMed database covering the period from 2020 to 2025 yielded relevant human studies on calcium, magnesium, iron, zinc, copper, selenium, and iodine in relation to depression, which were selected based on predefined inclusion and exclusion criteria. This review summarizes the effects of calcium, magnesium, iron, zinc, copper, selenium, and iodine on supporting prevention, slowing progression, and helping treatment of depression. Understanding the impact of proper nutrition, including ensuring optimal concentrations of minerals, can help develop dietary strategies or proper supplementation of macronutrients and micronutrients aimed at preventing and improving the functioning of patients with depression.

## 1. Introduction

Although the living environment seemingly provides all the resources necessary for maintaining good health, it appears to be a place where an increasing number of individuals are struggling with mental health issues. Depression is a common psychiatric disorder characterized by chronic, overwhelming sadness and anxiety; a loss of pleasure and interest in activities over a long period; sleep problems; and feelings of worthlessness [1]. However, the symptoms and severity of depression can vary from person to person. Year after year, the number of diagnoses rises, and patients experiencing depressive conditions at a younger age have been reported. It is estimated that approximately 280 million people worldwide suffer from depression. Additionally, this condition affects 50% more women than men [2]. Due to its high mortality rate among both young and elderly people, depression has become a global issue. For this reason, numerous studies have been conducted to explore the pathophysiology of this condition. Depression is a multifactorial disorder involving the dysregulation of several key signaling pathways. One of the main mechanisms involved in its pathophysiology is dysfunction of the hypothalamic–pituitary–adrenal (HPA) axis, resulting in impaired cortisol secretion and abnormal stress responses. Chronic activation of the HPA axis can harm neuroplasticity and hippocampal function [3]. Meanwhile, elevated levels of proinflammatory cytokines, such as IL-6, TNF-α, and IL-1β, indicate neuroinflammation, which can lead to impaired neurotransmitter synthesis and neuronal signaling [4]. Additionally, oxidative stress, resulting from an imbalance between reactive oxygen species and antioxidant defense systems, contributes to neuronal damage and mitochondrial dysfunction [5]. Dysregulation of neurotransmitters such as serotonin, dopamine, and norepinephrine affects mood, motivation, and cognitive function [6]. In light of the role of the mechanisms involved in the pathogenesis of depression, there is growing interest in the impact of nutritional factors, particularly minerals, on these biological systems.

**Figure 1:**
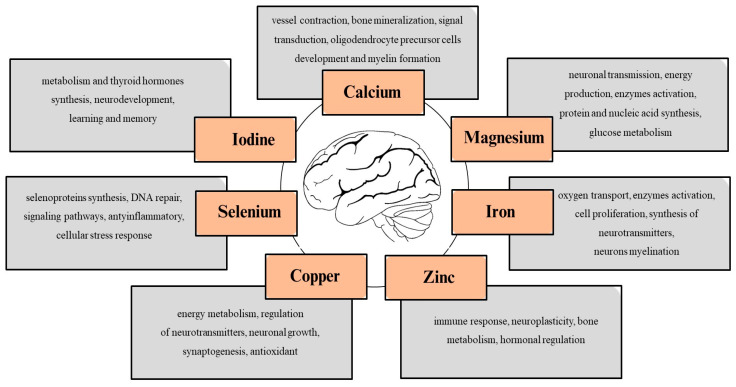
Minerals with protective effects on body and brain homeostasis [8,9,10,11].

Research indicates a correlation between dietary quality and mental health [7]. An adequate intake of macronutrients and micronutrients, such as calcium (Ca), magnesium (Mg), iron (Fe), zinc (Zn), copper (Cu), selenium (Se), and iodine (I), is essential for ensuring the physiological functioning of cells. These nutrients may exert a protective effect, or even slow down the progression of the disease, through various mechanisms [8,9,10,11]. For example, they can improve insulin sensitivity, modulate neurotransmission, minimize inflammation, and reduce free radicals (Figure 1). Therefore, a deficiency of these minerals in the daily diet may be associated with an increased risk of depression and other behavioral disorders. Understanding how these minerals act and their potential impact on the nervous system could encourage people to change their dietary habits out of concern for their mental health or as a complement to antidepressant treatment. We hypothesize that mineral imbalances may play an important, albeit under-recognized, role in the pathogenesis of depressive disorders by affecting neurotransmission, oxidative stress, and immune function.

This narrative review aims to provide a comprehensive assessment of the role of selected minerals—magnesium, iron, zinc, copper, selenium, and iodine—in the pathophysiology, prevention, and potential treatment of depressive disorders. The objective is to summarize the latest research on the impact of mineral imbalances on neurobiological processes such as neurotransmission, oxidative stress, and inflammation, and to evaluate whether interventions targeting mineral homeostasis, such as dietary changes or supplements, can support mental health and complement conventional depression therapies.

## 2. Methods

**Figure 2:**
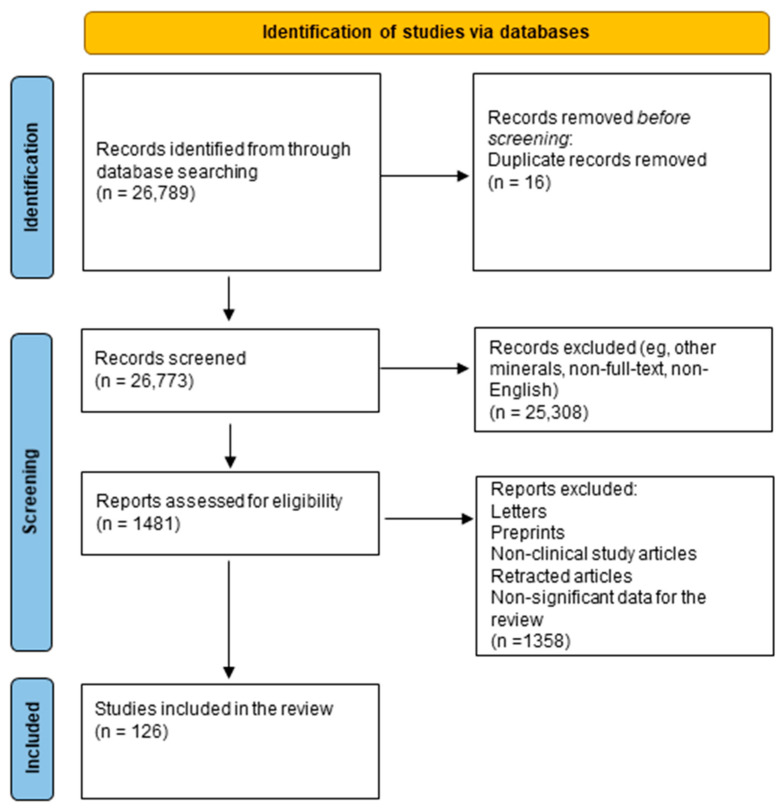
Prisma 2020 flow diagram depicting the methods for including studies in the review.

We have performed a comprehensive literature search in the MEDLAB/PubMed electronic database, covering the period from 2020 to 2025, using the keywords “minerals and depression” (n = 2565). The next step involved choosing macro- and microelements essential for the review: “calcium and depression” (n = 14,893), “magnesium and depression” (n = 2417), “iron and depression” (n = 2103), “zinc and depression” (n = 1914), “copper and depression” (n = 1174), “selenium and depression” (n = 585), and “iodine and depression” (n = 1138). Then, only publications in English, full-text, and limited to studies on humans were selected. In the next step, letters, non-clinical study articles, retracted articles, and non-significant data for the review were excluded (Figure 2).

## 3. Role of Macroelements in Depression

### 3.1. Calcium

Calcium ions play a role in various cellular processes, including contraction, differentiation, mitosis, cell death, and bone mineralization, thereby ensuring bone strength and structure [12,13,14]. In addition, as a second-order messenger, calcium is essential for signal transduction. It also plays a role in the immune response, acting as a secondary messenger in essential signal transduction pathways that activate, proliferate, and regulate immune cells, including T lymphocytes, B cells, and macrophages [15]. Recent studies have shown that calcium plays a modulatory role in the development of oligodendrocyte precursor cells and myelin formation [16]. The total Ca serum level ranges from 2.2 to 2.6 mmol/L, with over 90% localized in the bones [17]. The remainder circulates in the blood, either bound to proteins (40%) or organic anions (10%), or in its active ionized form (50%) [18]. Cytoplasmic and mitochondrial matrix calcium levels are typically lower than those in the extracellular fluid but may increase upon cellular stimulation [15]. Calcium absorption takes place in the intestines via passive and active transport, which is strongly influenced by calcitriol and the intestinal vitamin D receptor [19]. However, the amount absorbed decreases with age. Excessive calcium intake may contribute to pathological conditions such as nephrolithiasis, as well as vascular and soft tissue calcification [19]. Conversely, hypocalcemia can lead to tetany, cardiac dysfunction, or pre-eclampsia in pregnant women [15,20].

Interest in this nutrient in relation to depressive disorders stems from its capacity to influence the development and function of nervous tissue [16,21]. According to current knowledge, calcium ions may influence the extrapyramidal system by regulating the activity of D3 dopamine receptors present in this area. This affects the brain’s reward circuitry, cognitive functions, and emotional processing. The enzyme Ca^2+^/calmodulin-dependent protein kinase II (CaMKII), which is activated by the binding of calmodulin and calcium ions, phosphorylates the D_3_ receptor, thereby inactivating it [21]. This dependency may play a crucial role in maintaining dopaminergic homeostasis in the brain; disruption to this homeostasis is a significant factor in the pathophysiology of depressive disorders [22]. Interestingly, dysfunction of DR3 receptors has been associated with the development of mental disorders, including substance addiction, schizophrenia, and depression [21,23]. Additionally, CaMKII has been shown to regulate the activity of group I metabotropic glutamate receptors in the rat hippocampus, a mechanism that may contribute to the onset of chronic depression [24,25]. The important role of calcium in the development of mental disorders is also supported by studies on pregnant women, which indicate an inverse relationship between total serum calcium levels and the risk of depression. Women with lower calcium levels were found to be at a higher risk of developing depressive symptoms [26]. This dependence may be explained by the role of calcium ions in regulating the hypothalamic–pituitary–adrenal axis, which is considered the primary system responsible for the body’s response to stress [24]. Nevertheless, the exact mechanisms by which dietary calcium intake influences the development of depression remain unclear, and current findings are controversial [27].

### 3.2. Magnesium

Magnesium is the second most common intracellular cation in the human body after potassium. Its total mass equals around 25 g, 50–60% of which is situated in the bones [28]. The remainder is distributed in soft tissues, such as muscles [29]. As an intracellular ion, magnesium is involved in various biochemical reactions, including protein and nucleic acid synthesis, over 600 enzyme activations, neuronal transmission, and energy production [30]. As a natural calcium channel blocker, magnesium plays a crucial role in maintaining electrolyte balance and providing neuromuscular transmission by regulating sodium–potassium ATPase activity. Therefore, the proper concentration of Mg guarantees cellular membrane excitability [31]. Magnesium is essential for the proper functioning of the immune system. It regulates the activation of lymphocytes, maintains the stability of immune cell membranes, and modulates inflammatory responses by influencing the production of cytokines and oxidative stress [29]. Research also highlights its participation in glucose metabolism and tissue sensitivity to insulin [8]. The early signs of magnesium deficiency are not specific and include loss of appetite, nausea, vomiting, weakness, and lethargy [28]. Cardiac arrhythmias may also present as torsade de pointes, QT interval prolongation, or atrial and ventricular tachycardia [30]. Reduced magnesium levels may be caused by health conditions related to the digestive, renal, or endocrine systems or by poor dietary intake.

Hypomagnesemia is clinically defined as a serum magnesium concentration below 0.75 mmol/L [28]. Studies conducted in Europe and the United States consistently indicate that the general population does not consume the recommended daily amount of magnesium, highlighting a widespread dietary inadequacy [31,32]. However, magnesium deficiency often remains undetected as the symptoms are non-specific and the decline in serum levels is quickly compensated for by the release of magnesium from bone stores [31].

The literature indicates that magnesium plays a key role in modulating glucose transport across cell membranes and that low magnesium levels impair the tyrosine kinase activity of the insulin receptor. This leads to an increase in intracellular calcium concentration, which is associated with defective insulin signaling and reduced cell sensitivity to insulin [33]. Consequently, the patient develops insulin resistance (IR), as receptor excitability reduces and the autophosphorylation of the β-subunit is no longer effective [34]. If left untreated, insulin resistance stimulates the production of low-grade inflammatory cytokines, contributing to the development of type 2 diabetes. This, in turn, impairs brain dopamine and serotonin neurotransmission, which is an important factor in the emergence of depressive symptoms [35]. In a cohort group of 395 people diagnosed with T2D, the correlation between low plasma Mg^2+^ concentration and markers of insulin resistance was analyzed [36]. Evidence indicates that higher serum magnesium levels are associated with enhanced cellular sensitivity to insulin, reduced fasting glucose levels, and improved glycemic control in patients with type 2 diabetes [37,38]. Furthermore, it has been proven that prolonged hyperglycemia may influence the severity of depression and that a significantly lower magnesium concentration has been associated with an increased suicide rate in patients with depression [39,40]. Additionally, magnesium can cross the blood–brain barrier and is actively transported into the cerebrospinal fluid (CSF) with the participation of choroidal epithelial cells [41,42]. Other evidence-based research confirms that magnesium modulates neurotransmission involved in emotional processes, acting via the serotonergic, noradrenergic, dopaminergic, glutamatergic, and GABAergic systems [43]. This emerging modulation involves blocking the glutamatergic N-methyl-D-aspartate (NMDA) receptor in the central nervous system and intensifying the release of brain-derived neurotrophic factor (BDNF) in the hippocampus and amygdala, which suggests anxiolytic and antidepressant-like activity of Mg^2+^ [44]. Since the transport of calcium and sodium ions mainly occurs through NMDA channels, a magnesium deficiency may result in an excess of calcium current. This phenomenon is believed to disrupt neuronal function or even lead to neuronal death through the production of toxic reactive oxygen species or increased nitric oxide levels. These findings emphasize the importance of consuming magnesium-rich foods such as whole grains, beans, nuts, and green leafy vegetables daily. Maintaining a magnesium serum concentration within the reference range supports the brain’s cognitive functions by promoting neuron function, glucose metabolism, and the maintenance of brain monoamine neurotransmitters’ homeostasis. It may also enhance the effect of antidepressant treatment. Therefore, a magnesium deficiency has been suggested as a possible factor in the development of depressive disorders.

## 4. Role of Microelements in Depression

### 4.1. Iron

Iron is a trace element that is necessary for cellular functions such as respiration, oxygen transport, cell proliferation, and DNA replication. It is also crucial for immune function. It supports the proliferation and maturation of immune cells, particularly lymphocytes, and plays a key role in generating reactive oxygen species, which are used by macrophages to eliminate pathogens [45]. Many proteins involved in cellular metabolism and nucleic acid repair contain iron atoms in their molecular structure [46]. Furthermore, this trace element is crucial for the proper functioning of the central nervous system, neurotransmitter synthesis, and myelination [9]. The National Institutes of Health (NIH) states that the recommended daily intake of iron is 8 mg for men and 18 mg for women. During pregnancy, this increases to 27 mg/day (Table 1) [47]. 

Absorption of the micronutrient occurs in the duodenum and upper jejunum via enterocytes and the divalent metal transporter 1 (DMT1) [53]. Once in the blood, iron is bound by a protein called transferrin, which carries it to cells or bone marrow for use in erythropoiesis. Ferritin and hemosiderin are widely found in the body and act as storage media for iron [54]. Plasma iron levels and stored iron levels are controlled by the hormonal peptide hepcidin, which regulates iron absorption [46]. The most common disorder caused by insufficient iron consumption is iron deficiency anemia, which is clinically diagnosed based on blood count and iron metabolism results [47,55,56,57]. Symptoms indicating iron deficiency are often nonspecific, such as fatigue, exhaustion, an accelerated heartbeat, or a deterioration in emotional well-being [58,59]. Studies conducted on students have shown that iron levels affect cognitive function, with increased iron intake being correlated with a higher IQ [9]. In addition to deficiency, excessive iron intake can lead to various consequences, including oxidative DNA damage, decreased bone density, and an increased risk of cancer [60,61].

The symptoms of depression and iron deficiency are very similar [62,63]. The etiological factors contributing to both pathological conditions have been identified and include inflammation, degenerative changes, and oxidative stress [64]. Studies on women with anemia have shown a fourfold increase in the likelihood of developing postpartum depression, providing a basis for the hypothesis that iron may be involved in the development of affective disorders [59]. However, the precise biological mechanisms by which this trace element contributes to the disturbance of emotional homeostasis are not yet known. This makes it difficult to understand the etiology of depression and prevent its occurrence. Interestingly, iron deficiency is much more prevalent in women than in men [9,65]. According to the classical theory of depression, it is related to monoamine deficiency, including serotonin, dopamine (DA), and noradrenaline (NA), which influence emotional homeostasis. According to Berthou et al., iron acts as a cofactor of aromatic amino acid hydroxylases (phenylalanine, tyrosine, and tryptophan) [66]. The presence of these enzymes is essential for the proper synthesis of neurotransmitters such as dopamine, serotonin, and, indirectly, norepinephrine. As previously mentioned, NE increases the production of brain-derived neurotrophic factor [66]. The hippocampus, which is involved in cognition and emotions, appears to be particularly susceptible to a deficiency in BDNF, which is necessary for the proliferation and survival of neurons [67]. It has been proven that iron deficiency leads to a decrease in BDNF levels, which can increase the likelihood of depressive symptoms occurring [68]. Therefore, it can be expected that lowering serum iron levels contributes to ineffective neurotransmitter synthesis, resulting in disturbed neurotransmission in areas of the brain responsible for processing emotions. Under conditions of increased blood–brain barrier permeability, excess iron can enter brain tissue and have a degenerative effect, leading to neuroinflammation [69]. This phenomenon is evident in many neurodegenerative diseases, including Parkinson’s disease (PD), Alzheimer’s disease (AD), Huntington’s disease (HD), Friedreich’s ataxia (FA), and multiple sclerosis (MS). Iron overload in glial cells and neurons exacerbates the inflammatory process [69]. This mechanism is probably based on iron’s ability to induce oxidative stress, resulting in the excessive accumulation of reactive radicals, including the hydroxyl radical. These radicals are generated via the Fenton reaction, resulting in damage to DNA, proteins, and lipids, and consequently cell death [49].

### 4.2. Zinc

Zinc is a trace element that is essential for proper immune function, bone metabolism, and the regulation of hormones, including cortisol, as well as the growth of neurons. It is essential for immune function. It influences the development and activity of innate and adaptive immune cells, regulates the production of inflammatory cytokines, and helps to maintain the integrity of epithelial barriers [10]. The normal range for serum zinc in adults is 0.66–1.10 μg/mL [70]. Maintaining a balance between extracellular and intracellular zinc ions is extremely important for cell homeostasis in brain regions such as the hippocampus, amygdala, and cerebral cortex. Disruption to this balance may lead to depressive symptoms [70]. Zinc is also involved in the regulation of hormones, including cortisol, as well as in neuroplasticity and the cellular immune response. Zinc deficiency resulting from malnutrition can lead to hypogonadism, impaired ulcer healing, hair loss, and weight loss [71]. In the brain, zinc ions accumulate in the presynaptic vesicles of glutamatergic neurons; even subclinical deficiency of this element can therefore disrupt brain function. Conversely, excessive daily zinc supplementation can lead to copper deficiency or anemia [71].

Attention to zinc is growing due to its modulatory and anti-inflammatory properties. The literature widely describes the ability of zinc ions to decrease the expression of IL-1β, which belongs to the cytokine family and exhibits both pro-inflammatory and pro-apoptotic functions. Activation of procaspase 1 is necessary for these effects to occur [10]. Studies using zinc-containing compounds demonstrate that supplementation is associated with the degradation of the inactive precursor of caspase 1 (procaspase 1). It suggests a potential protective role for zinc against caspase-induced inflammation and neuronal apoptosis. This dependency has been supported by clinical research indicating increased IL-1β synthesis levels in overweight patients with poor daily zinc intake compared to controls with higher intake of this microelement [72]. This anti-inflammatory function could improve depressive patients’ sense of well-being and reduce the severity of their condition. As previously mentioned, chronic hyperglycemia contributes to the development and severity of depression [39]. Zinc deficiency impairs modulation of the ZnT8 transporter, leading to dysfunctional insulin storage and secretion and consequently to insulin resistance and increased inflammation. Interestingly, these insulin metabolism disorders have been proven to be reversible through zinc supplementation in animal studies [72]. It is assumed that an imbalance between the two key systems responsible for mental homeostasis, the excitatory (glutamatergic) and inhibitory (GABAergic) systems, plays a crucial role in depression [73]. According to this theory, excessive release of glutamate leads to hyperactivation of NMDA receptors, which zinc inhibits via two mechanisms: voltage-independent and non-competitive (allosteric) inhibition. This activity modulates neurotransmission in brain compartments responsible for processing emotions, and in some cases, zinc may act as a neuromediator itself [74]. Furthermore, NMDA receptors containing NR2A subunits are particularly sensitive to extracellular zinc ions [75,76]. Additionally, zinc influences mood improvement by intensifying brain-derived neurotrophic factors in the hippocampus and cortex [77]. Therefore, it is assumed that the recommended daily zinc intake is necessary for proper neurotransmission signaling and maintaining emotional homeostasis in humans. Studies conducted on mice have confirmed the involvement of zinc in antidepressant treatment. Animals lacking the G protein-coupled receptor (GPR39), which is activated by zinc, exhibit depressive behavior [76,78]. Additionally, the absence of the GPR39 receptor has been linked to reduced neuroplasticity and impaired 5-HT signaling, suggesting another potential mechanism underlying mental health conditions [73,76].

### 4.3. Copper

Copper is known to influence immune function, energy metabolism, the regulation of neurotransmitters, and neuronal growth [11]. It also plays a role in synaptogenesis, learning and memory, catecholamine metabolism, and the regulation of antioxidant processes. Unlike most other metals found in humans, copper (Cu) can accept and donate electrons, thereby changing its oxidation state between +2 (cuprous) and +1 (cupric) [11]. The total copper concentration in the human body ranges from 100 to 200 mg, and the recommended daily intake should not exceed 0.9 mg [79]. Additionally, copper acts as a cofactor for many biological enzymatic reactions. Copper deficiency may result in hematopoietic disorders, while excessive intake can lead to Wilson’s disease, which is characterized by the accumulation of copper ions in the body and causes damage to numerous cells [11]. Due to its ability to convert dopamine into norepinephrine, copper is considered to play a role in the development of depression [80].

Copper plays an important role in immune function. It supports the development and activity of neutrophils, macrophages, and T cells. It also participates in antioxidant defense through enzymes such as superoxide dismutase. This helps to regulate inflammation and protect against free radicals. Copper and zinc are essential cofactors of zinc-dependent superoxide dismutase 1 (SOD1), an enzyme that is present in large quantities in the cytosol and catalyzes the conversion of reactive superoxide, which is produced during mitochondrial respiratory chain reactions, into the less harmful hydrogen peroxide and water [81]. Copper deficiency may result in the accumulation of free radicals, particularly superoxide molecules, leading to cell damage and the initiation or intensification of inflammation. According to Huidan Deng et al., excessive exposure to copper atoms may also cause inflammatory responses by manipulating pathways such as the nuclear factor kappa-B (NF-κB) pathway, the mitogen-activated protein kinase (MAPK) pathway, the JAK-STAT pathway, and the NOD-like receptor protein 3 (NLRP3) inflammasome [82]. It is known that peripheral inflammation increases the permeability of the blood–brain barrier, resulting in disruption to brain homeostasis and leading to depression [83]. Another cause of barrier dysfunction may be an increased serum copper level, resulting in an increased concentration of the element in the brain [84]. Consequently, the accumulation of copper in brain tissue intensifies the catalysis of reactive oxygen species (ROS), thereby increasing the neurotoxic effects of oxidative stress and causing neuronal damage. This may contribute to the development of depression [85,86,87].

The adrenal gland and noradrenergic neurons are key sources of catecholamines, including norepinephrine (noradrenaline) and epinephrine (adrenaline). Noradrenergic neurons primarily produce norepinephrine (NE) through the action of dopamine-β-hydroxylase (DBH), an enzyme that hydroxylates dopamine using two uncoupled copper atoms [81]. In contrast, the adrenal medulla produces both norepinephrine and epinephrine. The final step in this process, the conversion of norepinephrine to epinephrine, is catalyzed by phenylethanolamine N-methyltransferase (PNMT). This enzyme is predominantly expressed in adrenal chromaffin cells. Norepinephrine functions mainly as a neurotransmitter in the central and peripheral nervous systems, while epinephrine acts primarily as a hormone that is released into the bloodstream during acute stress. Norepinephrine is one of the main catecholamines implicated in the monoamine hypothesis of depression, influencing arousal, mood regulation, and the stress response [76]. Research investigating the relationship between daily copper consumption and the occurrence of depressive symptoms in adults has indicated a negative correlation between high serum copper levels and the incidence of depression [80]. This phenomenon may be initiated by excessive noradrenaline synthesis under conditions of increased dopamine conversion, resulting in the arousal of the sympathetic nervous system at the synaptic level. Noradrenergic dysregulation can lead to an increased perception of anxiety, weakened neuroplasticity, and reduced cerebral blood flow [88]. Studies performed on postpartum women suffering from affective disorders also exhibit elevated serum Cu levels compared to non-depressive controls [89]. Increased copper intake may result in the dysregulation of catecholamine neurotransmitters, as well as in cytotoxicity and cuproptosis, which can lead to cell death, including that of neurons [79]. This process is initiated by the binding of Cu^2+^ ions with nitrogen (N) and sulfur (S) atoms, causing rapid transport of Cu^2+^ into the mitochondria. In this process, Cu^2+^ ions are reduced to Cu^1+^ forms and reactive oxygen species are released [79]. Subsequently, the binding of Cu^2+^ ions to the acylated components ensures the aggregation of lipid-acylated-related proteins. Additionally, loss of iron–sulfur cluster proteins is observed, leading to cell death. Therefore, it can be assumed that depression may develop if areas of the brain that are important for maintaining emotional homeostasis, such as the limbic system or amygdala, are involved in such processes.

### 4.4. Selenium

Selenium is essential for optimal immune function. It contributes to the body’s antioxidant defenses through selenoproteins. It also modulates cytokine production and supports the activation and proliferation of T cells and natural killer cells. This enhances the body’s ability to respond to infections and inflammation. To date, 25 selenoproteins have been identified in the human genome, including glutathione peroxidase (GPx), thioredoxin reductase (TXNRD), and iodothyronine deiodinases (DIOs) [90]. These molecules are particularly known for their roles in suppressing inflammatory processes, responding to cellular stress, repairing DNA, and maintaining protein quality control. Thus, an appropriate daily intake of bioavailable selenium is crucial for the proper functioning of nearly all human cells, particularly neurons in the central nervous system and skeletal muscles, where most of this element is stored, as well as the immune and endocrine systems [90]. Currently, an optimal daily selenium intake of 55 μg/day is recommended [91]. However, studies report that the form of selenium consumed (organic or inorganic) may be more important than the dosage [90]. It is worth mentioning that both selenium deficiency and excessive consumption (selenosis) can lead to health deterioration. For instance, selenium deficiency contributes to male infertility and Keshan disease, whereas selenosis can result in kidney and liver damage [92].

Several hypotheses regarding the protective effects of selenium on mental health have been analyzed. The ability to modulate multiple brain signaling pathways, including the serotonergic, dopaminergic, and noradrenergic systems, has sparked particular interest in this microelement [91]. In addition to the functions above, selenium’s antagonistic effect towards glutamine toxicity, interaction with redox signaling mechanisms, inflammatory modulation, and participation in neuronal metabolism have also been noted [93]. Notably, the thyroid has the highest concentration of selenium among all endocrine organs. This phenomenon is presumably connected to its participation in thyroid hormone metabolism. Metabolically active triiodothyronine (T3) is created through the conversion of thyroxine (T4), and selenium is incorporated into iodothyronine deiodinases, which catalyze this process [91]. A serum concentration of these molecules within the reference range is essential for proper nervous system development, and decreased levels affect mood, a phenomenon that is attracting increasing attention [94]. Recent studies have shown that depressive symptoms in patients are negatively correlated with triiodothyronine and thyroxine serum levels and positively correlated with TPOAb concentration [95]. This phenomenon may be caused by insufficient stimulation of the limbic system, which is located in the brain and is responsible for cognitive functions, memory, and emotional processing. Interestingly, this system’s surface is densely lined with receptors for thyroid hormones, whose synthesis depends on selenium concentration. Therefore, hypothyroidism, a health condition characterized by decreased thyroxine and triiodothyronine secretion, may lead to depression by reducing stimulation of the aforementioned limbic system [95]. The literature draws attention to the anti-inflammatory effect of selenium, as blocking the synthesis of certain cytokines by this element has been proven [91]. For example, high levels of CRP and interleukin-6 in the serum strongly stimulate inflammatory processes and have been demonstrated to be inversely correlated with serum Se concentrations. Additionally, glutathione peroxidase protects thyrocytes against the accumulation of excess hydrogen peroxide (H_2_O_2_), preventing damage [96]. Selenium indirectly stimulates the limbic system and the expression of brain-derived neurotrophic factor, thereby promoting emotional balance. BDNF ensures neuronal network plasticity, and decreased levels have been observed in cases of depression. The relationship between a decline in BDNF and the concentrations of other neurotrophic factors impairs synaptic transmission and neurogenesis, which may result in a deterioration in mood, experience, and sense of well-being [91]. Although the expression of BDNF in humans is not yet well understood, a significant reduction in this factor was observed in rats under conditions of selenium deficiency. Furthermore, recent studies by Lee et al. indicate that depression is present in postpartum women who exhibit significantly lower BDNF concentrations than healthy pregnant women [97].

Given selenium’s wide range of protective functions for brain homeostasis, antidepressant therapy supported by supplementation of this micronutrient may improve treatment outcomes.

### 4.5. Iodine

Iodine primarily influences immune function through its role in the synthesis of thyroid hormones, which regulate the maturation and activity of immune cells. Additionally, iodine and its compounds possess antimicrobial properties and can modulate inflammatory responses [98]. Iodine is a trace element that also plays a key role in neurodevelopment in fetuses and children. It also supports overall metabolism and mental competence, including learning and memory. Most importantly, it is essential for the functioning of the thyroid gland, as it is a key component of thyroid hormones such as thyroxine and triiodothyronine. These hormones affect brain development, mood, and cognitive functions [98,99,100]. According to the National Institutes of Health (NIH), the recommended daily iodine intake is 120 µg for children and 150 µg for adults [52]. Iodine deficiency during pregnancy can lead to preventable intellectual disability, impaired growth and thyroid hormone synthesis, and damage to the central nervous system (Table 2) [101,102]. Therefore, pregnant women should consume at least 220 µg of iodine per day. The adult human body contains around 15–20 mg of iodine, 70–80% of which is localized in the thyroid gland. When dietary iodine is sufficient, thyroid iodine uptake remains low, typically around 10%, but in cases of chronic deficiency, it can rise to over 80% [98]. Iodine deficiency in the diet can lead to a condition called hypothyroidism, which is more prevalent in women than in men [103]. Symptoms include weariness and anxiety and can be associated with depression [104]. Furthermore, untreated thyroid dysfunction not only results in mood disorders such as depression but also infertility, cardiovascular disease, and neurological and musculoskeletal symptoms [105,106].

The literature states that hypothyroidism and altered hypothalamic–pituitary–thyroid axis functioning may be associated with depression [107]. While the exact mechanism is unclear, it is hypothesized that disruption to this pathway reduces the accessibility of thyroid hormones, thereby impairing synaptic plasticity and the myelination of neurons within the brain [108]. Decreased plasticity and impaired myelination have both been described as contributing to affective disorders [109]. Due to the crucial role of thyroid hormones in regulating cerebral metabolism and neuroendocrine function, thyroid dysfunction has been associated with the development of postpartum and perinatal depression [110]. However, the relationship between habitual dietary iodine intake during pregnancy and associated health outcomes remains insufficiently studied, with limited data currently available. One study of pregnant women with mild-to-moderate iodine deficiency found that lower dietary iodine intake during the second trimester was associated with higher levels of perinatal and six-month postpartum depressive symptoms [103]. Paradoxically, iodine supplementation was also linked to an increase in postpartum depression scores, indicating a potentially complex, dose-sensitive relationship between iodine intake and maternal mental health. According to Wang et al., no significant differences were found in depression scores or thyroid-stimulating hormone (TSH) levels one month postpartum [111]. However, they reported a difference in free thyroxine (FT4) levels between the group that received iodine supplements (150 µg/day) and those who received supplements without iodine or no supplements at all. Depression scores were higher in the iodine supplement group, although the difference did not reach statistical significance [111].

**Table 2:** Health effects of deficiency and excessive intake of selected macro- and micronutrients.

Element	Deficiency	Excess	References
Calcium	Osteopenia, osteoporosis, muscle cramps, rickets in children, impaired nerve transmission, and hypertension	Hypercalcemia, kidney stones, impaired absorption of other minerals (e.g., iron and zinc), and constipation	[15,19,20,26]
Magnesium	Muscle weakness, tremors, arrhythmias, fatigue, irritability, and increased risk of depression	Diarrhea, hypotension, nausea, and cardiac arrest	[28,30,31,34,37,38]
Iron	Anemia, weakened immunity, and developmental delays	Hemochromatosis, liver damage, oxidative stress, increased risk of infections, and cardiovascular disease	[47,55,56,57,58,59,60,61]
Zinc	Impaired immune function, growth retardation, hair loss, diarrhea, delayed wound healing, and taste disturbances	Nausea, vomiting, immune suppression, copper deficiency, and impaired HDL levels	[71,72]
Copper	Anemia, neutropenia, bone abnormalities, neurological symptoms (e.g., ataxia), and connective tissue defects	Liver damage, gastrointestinal distress, neurotoxicity, and Wilson’s disease	[11,79,82]
Selenium	Hypothyroidism, fatigue, infertility, impaired immunity, and cardiomyopathy (Keshan disease)	Selenosis, gastrointestinal upset, and nervous system abnormalities	[90,91,92]
Iodine	Goiter, hypothyroidism, impaired cognitive development, cretinism in infants, and reproductive dysfunction	Hyperthyroidism, thyroid inflammation, iodine-induced goiter, metallic taste, and skin lesions	[50,51,101,102,103,104,105,106,107,108,109,110,111]

## 5. Limitations and Future Research Directions

Other interesting issues include the interaction between mineral metabolism and the gut microbiome, sex-specific differences, and epigenetic mechanisms. The gut microbiota plays a pivotal role in the absorption, bioavailability, and regulation of macronutrients and micronutrients. Dysbiosis can impair nutrient absorption and modulate inflammatory and neuroimmune responses, which may be associated with depression. There is emerging evidence that gut dysbiosis can impair calcium uptake, induce inflammation, and indirectly contribute to the activation of neuroinflammatory pathways associated with depression. Furthermore, changes in microbial composition, such as shifts in the *Firmicutes/Bacteroidetes* ratio, can impact skeletal health and various neuroendocrine functions [112]. Furthermore, a study of over 1700 mother–child pairs revealed that calcium supplementation during the early years of life was linked to a higher prevalence of beneficial gut bacteria, particularly *Lactobacillus* [113]. The study of Jamilian et al. evaluated the effect of simultaneous administration of probiotics and selenium on mental health in women with polycystic ovary syndrome (PCOS). After 12 weeks of supplementation, there was a significant improvement in depression test results and overall health, as well as a reduction in anxiety and stress compared to the placebo group [114]. Future research should investigate how personalized regulation of the gut microbiome—through diet, prebiotics, or probiotics—can improve mineral homeostasis and thereby support mental health. Combining gut microbiome analysis with nutrient status and psychiatric assessment could lay the groundwork for developing personalized interventions to support therapy for mood disorders [115].

In addition, differences in mineral requirements between the sexes, as well as the influence of hormones on mineral homeostasis, may affect men’s and women’s susceptibility to mood disorders differently. Gender differences in the metabolism of macronutrients and micronutrients may affect nutrient requirements, absorption, and distribution. These differences can also be attributed to earlier skeletal maturation in women or higher bone mass in men, both of which affect calcium retention and bone mineral requirements. By contrast, premenopausal women have greater iron requirements due to blood loss during menstruation, while mineral intake during pregnancy may affect fetal growth and development. Furthermore, emerging data suggest that hormonal fluctuations, particularly those involving estrogen and testosterone, may modulate mineral metabolism and inflammatory pathways differently in men and women [116]. Therefore, future studies should consider sex differences as a biological variable when designing dietary or supplementation strategies.

Finally, epigenetic modifications, such as DNA methylation and histone acetylation, can regulate genes involved in mineral transport and metabolism. Iron homeostasis, for example, is tightly controlled by a network of genes involved in absorption, transport, storage, and cellular utilization (DMT1, TFR1, TFR2, FPN, HAMP, and ferritin H). Recent findings suggest that epigenetic factors such as DNA methylation, histone modifications, and microRNAs (miRNAs) can significantly influence the expression of these genes. Hypermethylation of promoter regions in key iron-related genes such as FPN, HAMP, and HJV has been shown to repress transcription, while histone deacetylase (HDAC) activity downregulates HAMP expression—an effect that can be reversed by HDAC inhibitors. Additionally, specific miRNAs have been found to post-transcriptionally regulate several iron transport and storage genes, thereby influencing iron availability [117].

Future research should focus on identifying reliable mineral metabolism-related biomarkers that could aid in the early detection of depressive disorders and the monitoring of treatment responses. Another promising area of research is the development of personalized supplementation strategies based on mineral content, gut microbiota status, and genetic predisposition. Tailoring interventions to the specific nutritional and physiological needs of patients by combining targeted mineral supplementation with conventional pharmacological treatments may enhance therapeutic efficacy, particularly in treatment-resistant cases. This approach can also minimize the risks associated with excessive supplementation.

This review highlights the potential links between mineral homeostasis and depressive disorders; however, several important limitations should be noted. Most of the reviewed studies are observational, which restricts the ability to draw definitive conclusions that support a fundamental role for mineral imbalance in depression. The mechanisms by which minerals affect mood regulation are not fully understood, particularly in the context of neuroinflammation and hormonal regulation. Future studies should consider factors such as gender, age, microbiome composition, and epigenetic background to improve our understanding of the role of individual minerals in the pathophysiology and treatment of depression.

## 6. Conclusions

In conclusion, this narrative review shows that imbalances in minerals such as magnesium, iron, zinc, copper, selenium, and iodine could lead to depressive disorders by causing oxidative stress, neuroinflammation, and changes in neurotransmission. The literature has extensively documented these mechanisms, suggesting the potential of minerals as adjuncts to antidepressant therapy (Table 3). 

However, the current body of evidence does not support their use as stand-alone treatments, in part because of variability in study design, dosing, population characteristics, comorbidities, and dietary context. While numerous studies have highlighted the beneficial effects of these minerals on cognition and mood regulation, the inconsistencies in the results underscore the need for high-quality, well-controlled studies. A balanced, nutrient-rich diet, along with lifestyle changes, may be a promising strategy to support mental health and improve quality of life in individuals at risk for or living with affective disorders. Further research is needed, including interventional studies, but these findings highlight the importance of considering individual mineral status in clinical practice and when developing adjunctive therapeutic strategies for mood disorders.

## Figures and Tables

**Table 1:** List of all recommended daily intakes and dietary sources related to the minerals discussed.

Minerals	Symbol	Recommended Daily Intake			Dietary Sources	References
		Age	Male	Female		
Calcium	Ca	1–3 years	700 mg	700 mg	milk, yogurt, cheese, sardines, salmon, kale, and broccoli	[17]
		9–13 years	1300 mg	1300 mg		
		19–50 years	1000 mg	1000 mg		
Magnesium	Mg	1–3 years	80 mg	80 mg	spinach, legumes, nuts, seeds, and whole grains	[12]
		9–13 years	240 mg	240 mg		
		31–50 years	420 mg	320 mg		
Iron	Fe	1–3 years	7 mg	7 mg	nuts, beans, vegetables, and fortified grain products	[48]
		9–13 years	8 mg	8 mg		
		19–50 years	8 mg	18 mg		
Zinc	Zn	1–3 years	3 mg	3 mg	meat, fish, seafood, and eggs	[49]
		9–13 years	8 mg	8 mg		
		19+ years	11 mg	8 mg		
Copper	Cu	1–3 years	340 µg	340 µg	shellfish, seeds, nuts, organ meats, wheat-bran cereals, whole-grain products, and chocolate	[50]
		9–13 years	700 µg	700 µg		
		19+ years	900 µg	900 µg		
Selenium	Se	1–3 years 9–13 years 19–50 years	20 µg 40 µg 55 µg	20 µg 40 µg 55 µg	Brazil nuts, seafood, meat, poultry, organ meats cereals, and eggs	[51]
		9–13 years	40 µg	40 µg		
		19–50 years	55 µg	55 µg		
Iodine	I	1–3 years	90 µg	90 µg	seaweed (kelp, nori, kombu, and wakame), fish, and eggs	[52]
		9–13 years	120 µg	120 µg		
		19+ years	150 µg	150 µg		

**Table 3:** Mineral supplementation in the therapy of depression.

Mineral	Key Findings	Reference
Magnesium (Mg)	A magnesium dose of 248 mg/day improved depression symptoms in 6 weeks, comparable to selective serotonin reuptake inhibitors in mild–moderate cases.	[118]
	Administration of 500 mg of magnesium per day can improve the depression status in adults.	[119]
	Vitamin D plus magnesium supplementation in obese women with mild to moderate depressive symptoms has beneficial influences on mood, serum levels of brain-derived neurotrophic factor, inflammation, and sirtuin 1.	[120]
Iron (Fe)	Supplementation improved fatigue and mood in women with iron deficiency.	[121]
Zinc (Zn)	Supplementation reduced depressive symptoms, may enhance antidepressant efficacy, and is associated with increased brain-derived neurotrophic factor levels.	[122]
	Zinc supplementation, together with selective serotonin reuptake inhibitors antidepressant drugs, improves major depressive disorders more effectively in patients with placebo plus antidepressants (selective serotonin reuptake inhibitors).	[123]
	The use of zinc supplements improved depression and anxiety in the elderly.	[124]
Selenium (Se)	Potential protective effect against depression—the lower the level of selenium in the diet, the more reports of anxiety, depression, and tiredness decreased following 5 weeks of selenium therapy.	[125]
	Supplementation with selenium during pregnancy might be an effective approach for the prevention of postpartum depression.	[126]
